# Decision-making processes for essential packages of health services: experience from six countries

**DOI:** 10.1136/bmjgh-2022-010704

**Published:** 2023-01-19

**Authors:** Rob Baltussen, Omar Mwalim, Karl Blanchet, Manuel Carballo, Getachew Teshome Eregata, Alemayehu Hailu, Maryam Huda, Mohamed Jama, Kjell Arne Johansson, Teri Reynolds, Wajeeha Raza, Jacque Mallender, Reza Majdzadeh

**Affiliations:** 1 Department of Health Evidence, Radboudumc, Nijmegen, The Netherlands; 2 Department of Global Public Health and Primary Care, University of Bergen, Bergen, Norway; 3 Global Health Development, University of Geneva Faculty of Medicine, Geneve, Switzerland; 4 International Centre for Migration and Health, Geneva, Switzerland; 5 Department of Global Public Health and Primary care, University of Bergen Faculty of Medicine and Dentistry, Bergen, Norway; 6 Harvard University T H Chan School of Public Health, Boston, Massachusetts, USA; 7 Department of Community Health Sciences, Aga Khan University Hospital, Karachi, Pakistan; 8 Federal Government of Somalia, Mogadishu, Somalia; 9 Department of Integrated Health Services, World Health Organization, Geneva, Switzerland; 10 Centre for Health Economics, University of York, York, UK; 11 EconomicsbyDesign, London, UK; 12 School of Health and Social Care, University of Essex, Colchester, UK

**Keywords:** Public Health

## Abstract

Many countries around the world strive for universal health coverage, and an essential packages of health services (EPHS) is a central policy instrument for countries to achieve this. It defines the coverage of services that are made available, as well as the proportion of the costs that are covered from different financial schemes and who can receive these services. This paper reports on the development of an analytical framework on the decision-making process of EPHS revision, and the review of practices of six countries (Afghanistan, Ethiopia, Pakistan, Somalia, Sudan and Zanzibar-Tanzania).

The analytical framework distinguishes the practical organisation, fairness and institutionalisation of decision-making processes. The review shows that countries: (1) largely follow a similar practical stepwise process but differ in their implementation of some steps, such as the choice of decision criteria; (2) promote fairness in their EPHS process by involving a range of stakeholders, which in the case of Zanzibar included patients and community members; (3) are transparent in terms of at least some of the steps of their decision-making process and (4) in terms of institutionalisation, express a high degree of political will for ongoing EPHS revision with almost all countries having a designated governing institute for EPHS revision.

We advise countries to organise meaningful stakeholder involvement and foster the transparency of the decision-making process, as these are key to fairness in decision-making. We also recommend countries to take steps towards the institutionalisation of their EPHS revision process.

**Summary box**

- Reviewed countries use a similar stepwise approach in organising their decision-making process on essential packages of health services (EPHS) revision, but differ in the way they organise the specific steps.
- To foster fairness of decision making, we advise countries to ensure meaningful stakeholder involvement and be transparent throughout the entire decision-making process.
- In order to have a lasting impact, we advise countries to institutionalise their decision-making process on EPHS revision by establishing a legal framework, creating an adequate governance structure, and allocating sufficient analytical and financial capacity.
- Countries can learn from international experience on revising their EPHS, but they should tailor their revision process according to their own decision-making context.

## Background

 Many countries around the world strive for universal health coverage (UHC), to provide the health services their populations need without causing financial hardship. An essential packages of health services (EPHS) is a central policy instrument for countries to achieve this, as it defines the coverage of services that are made available, as well as the proportion of the costs that are covered from different financial schemes and who can receive these services. Such EPHS can guide both the delivery of care and the associated resource allocation, including human resources, provider payment, procurement and budgeting.^1^,^3^

Traditionally, analytical work to support EPHS revision has placed emphasis on evidence and analysis of themes such as effectiveness, safety, cost, cost-effectiveness (CE), burden of disease and budget impact of health services.^3^ Only recently attention is being paid to the process of EPHS revision. The way a country organises its decision-making process can have far-reaching consequences for the contents, fairness and impact of its EPHS.^4^,^12^

This paper reviews the experience of six countries (Afghanistan, Ethiopia, Pakistan, Somalia, Sudan and Zanzibar, a semiautonomous part of Tanzania) in terms of how they organised their decision-making process. The selection of countries was based on their use of Disease Control Priorities 3 (DCP3)-related evidence in EPHS revision,^13^ and subsequent involvement in the DCP3 Country Translation Review Initiative. For the review, we developed an analytical framework and a country information template (online supplemental box S1) on the decision-making process for EPHS revision. This was based on intensive discussions using several review rounds among all authors, with reference to guides relevant to EPHS revision.^5^,^11^ Informants were persons leading and involved in the management of EPHS development or revision in the six countries during the period 2019–2022. We also developed general recommendation on how countries can improve their current EPHS revision process, on the basis of review results, discussions among authors and available sources on EPHS revision.^5^,^11^ In doing so, we do not provide a blueprint for EPHS revision and recognise that countries will have their own decision-making process.

Countries differ in their institutional arrangements regarding EPHS revision, and in this paper, we use the term ‘governing body’ when we refer to the principal agency governing the EPHS, for example, the Ministry of Health or an agency external to it. We interchangeably also refer to ‘countries’, and this relates to governing bodies in countries. Wherever we use the term ‘EPHS revision’, it may also refer to EPHS design if a country is yet to establish its EPHS.

## Analytical framework

**Figure 1:**
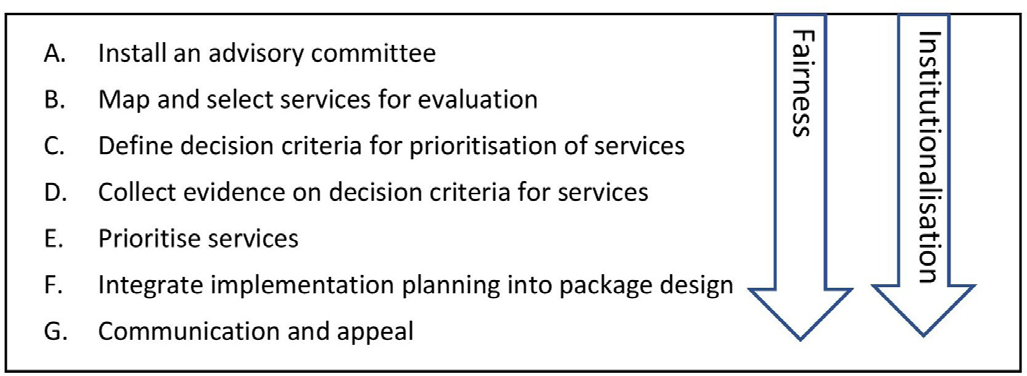
The stepwise EPHS revision process. EPHS, essential packages of health services.

The analytical framework distinguishes three interrelated topics of a country’s decision-making process: practical organisation, fairness and institutionalisation (figure 1).

In order to address practical organisation, we developed a seven-step EPHS revision process, informed by several sources^5^,^11^ and the experience in the six countries around questions such as: what evidence must be collected, for which services, who should decide which services to include, on what basis and how to take the current health system into account.

The fairness of EPHS revision refers to the reasonableness of decisions as perceived by domestic stakeholders, and this is an important requisite for societal support for the final EPHS.^6 14^ There is a growing acknowledgement of the need for decision makers to organise processes that are fair and to do so in a pragmatic manner.^6 14^ We use the evidence-informed deliberative processes’ framework, which distinguishes four elements that countries can use in each step of their decision-making process to foster the fairness of their process: meaningful stakeholder involvement, ideally operationalised through deliberation; evidence-informed evaluation; transparency and appeal.^15^

Institutionalisation is defined as how a set of activities becomes an integral part of a planning system and is embedded in ongoing practices.^11 16^ Countries may want to institutionalise the decision-making process so as to facilitate any ongoing EPHS revision and realise a lasting impact on the EPHS.^11 16^ The institutionalisation of EPHS revision relates to issues such as legal framework, governance and capacity.

Below we describe the seven steps of the EPHS decision-making process, and provide for each step review results of how the six countries implemented these steps. We also provide review results for the topic of Institutionalisation. Our general recommendations are listed in box 1.

Box 1 **Recommendations on the implementation of the essential packages of health services (EPHS) decision-making process**

**Step A: instal an advisory committee**

1. Have a governance structure in place that clearly describes the roles and mandates of the various institutions and stakeholders involved.
2. Instal an ‘advisory committee’ whose main task is to prepare recommendations on EPHS revision to the final decision-maker.
3. Instal ‘technical task forces’ that can support the advisory committee.
4. Compose the advisory committee in a way that it reflects the diversity of social values present in the population, and involve, in addition to health experts, non-health professionals.
5. Describe the membership and recruitment process of the advisory committee in a publicly available document.
6. Actively involve all relevant stakeholders in the decision-making process—this can be done through participation, consultation or communication.

**Step B: map and select services for evaluation**

1. Assess which model package (such as the DCP3 HPP or EUHC) is most relevant to the decision-making context.
2. Assess the relevance of included services vis-à-vis the sociocultural and epidemiological context and compare the resulting list of services with the existing package.
3. Make a choice whether to evaluate all services in detail or only concentrate evaluation activities on selected set of services.
4. Involve stakeholders in the selection of services and describe the process in a publicly available document.

**Step C: define decision criteria for prioritisation of services**

1. Define decision criteria in consultation with stakeholders and consider their values.
2. Describe the decision criteria and their selection process in a publicly available document.

**Step D: collect evidence on decision criteria for each service**

1. Organise an independent review of quality of evidence by stakeholders and experts.
2. Make the used evidence available publicly.

**Step E: prioritise services**

1. Present evidence in a way that is easily accessible and understandable by the advisory committee.
2. Use a structured approach to interpret this evidence and to trade-off decision criteria, such as qualitative, quantitative or decision rules analysis.
3. Always include a deliberative component in this structured approach to secure the quality of the decision.
4. Involve stakeholders in the prioritisation of services.
5. Describe the prioritisation process in a publicly available document, and report on the deliberations and the underlying argumentation for specific decisions.

**Step F: integrate implementation planning into EPHS revision**

1. Establish a plan that describes how services are implemented in terms of various health system aspects such as copayments, delivery platform, health system barriers and required investments.
2. Secure an integrated service delivery, that is, include foundational services for undifferentiated conditions in the package and coordinate services across different levels of the health system to foster continuity of care.
3. Develop the implementation plan in conjunction with stakeholders and make it publicly available.

**Step G: communication and appeal**

1. Ensure that EPHS coverage decisions are communicated to all relevant stakeholders, using a variety of channels.
2. Establish a protocol for appeal, including the requirements regarding provision of new evidence and clear revision rules.

**Institutionalisation**

1. Institutionalise the decision-making process for ongoing EPHS revision.
2. Establish an explicit requirement, for example, legal framework that ensures ongoing EPHS revision.
3. Designate an institution for governing ongoing EPHS revision.
4. Describe the EPHS revision process in a formal document.
5. Secure sufficient funds for EPHS revision.
6. Secure sufficient technical capacity for EPHS revision and make plans to improve capacity when insufficient.
  DCP3, Disease Control Priorities 3; EUHC, essential universal health coverage; HPP, high priority package.

## Step A: install an advisory committee

### Advisory committee

Countries can instal an advisory committee, that is, a central decision-making committee that prepares recommendations on EPHS revision for consideration by the final decision-maker, typically the Ministry of Health.^11^ In the development of these recommendations, the committee makes scientific and social judgments on the coverage of services, costs and populations in the EPHS.^4^ To avoid cognitive overload, the advisory committee can be supported by subcommittees that develop preparatory recommendations on specific disease programmes. The governing body may also wish to instal technical task forces that can provide assistance to the advisory committee, for example, in terms of evidence collection.^4^ Our analysis shows that all the six countries had an advisory committee in place, and that these were often assisted by subcommittees and some form of technical support (online supplemental table S1).

### Stakeholder involvement

Given that the advisory committee informs public decision making, it is generally advised that its members should ideally reflect the needs and interests of the broader public.^11^ This means that the composition of the committee should mirror the demographic and social diversity of the population and its social values, needs and preferences, and can involve both health experts (such as clinicians, public health professionals, programme managers and patients’ organisations) and non-health professionals (such as community members, policy makers, politicians, researchers, development partners and civil society).^17^ Here, the critical need for and the value of involving community representatives in advisory committees is often neglected.

Such stakeholders can be involved in decision making in three different ways^18^: (1) they can participate in meetings and engage in deliberations with or without voting; (2) they can be consulted, that is, involved in non-deliberative ways, such as through the provision of verbal comments at meetings and (3) they can be involved through stakeholder communication in which stakeholders are only informed about the processes and/or decisions.

Our review showed that in four of the focus countries (Afghanistan, Ethiopia, Pakistan and Zanzibar), advisory committees and subcommittees involved stakeholders such as health professionals, provincial representatives and development partners. In Zanzibar, patient representatives and people from within the community were also involved. Stakeholders actively participated in deliberations in all countries, with stakeholders in Pakistan also having voting rights.

### Conflict of interest and transparency

The advisory committee is ideally independent and free of undue external influences.^10^ It is therefore important that advisory committees do not include stakeholders who have interests in specific services.^10^ If potential conflicts of interests do exist, these can be openly declared^19^ (as was the case in Pakistan) and appropriate steps can be taken to resolve conflicts if and when any are identified. Countries can describe the membership and recruitment process in publicly available documents, as was done in most of the six countries (Afghanistan, Pakistan, Sudan, Zanzibar), and typically by means of a written report. In Somalia, this information was proactively sent to stakeholders.

## Step B: map and select services for evaluation

Countries can use model packages as a starting point for their EPHS revision—these describe a set of services that a typical country may want to include in its EPHS. Central to DCP3 are (A) the high priority package (HPP) which includes 108 services and is most relevant for low-income countries and (B) the essential universal health coverage (EUHC) package, which includes 218 services and is most applicable to lower-middle-income countries.^13^ However, countries may wish to combine these packages with other recommended packages or listings of services such as the UHC Compendium,^20^ in order to have a more comprehensive starting point for analysis. Our review shows that countries used various packages as the starting point of analysis (online supplemental table S2). Three countries (Pakistan, Somalia and Sudan) used the DCP3 EUHC package, Somalia added services from the UHC Compendium reflecting the need to cover services for common symptomatic presentations, and Sudan added services from the WHO-Eastern Mediterranean Region UHC Priority Benefit Package. Afghanistan used the HPP as a starting point for its analysis.

Countries can involve stakeholders in the selection of services for evaluation, and describe the process in a publicly available document.^10^ In most countries, stakeholders were involved through membership in (sub) committees. Two countries (Afghanistan, Somalia) made information on the selection of services public.

## Step C: define decision criteria for prioritisation of services

Decision criteria reflect the broad goals of a country’s health system (eg, maximisation of population health, fair distribution of health and financial protection) and underlying values (eg, equity, solidarity and access to good quality care).^11 21^ The advisory committee can use decision criteria for the assessment and subsequent appraisal of services, and in this way, recommendations on the inclusion or exclusion of services in the package of essential health services are based on social preferences. Countries are generally advised to define such decision criteria in consultation with stakeholders and to take into account their different needs, interests and values.^11^ There are various ways to organise such a consultation, for example, through policy document review, survey or a workshop. Countries can publish decision criteria in a publicly available document.

Our analysis showed that countries most frequently used CE as a criterion (Ethiopia, Pakistan, Somalia, Sudan and Zanzibar), followed by financial risk protection (FRP) and equity (Afghanistan, Ethiopia, Pakistan and Zanzibar), and budget impact (Ethiopia, Pakistan, Somalia and Sudan) (online supplemental table S3). Less commonly used decision criteria concerned feasibility/health system capacity (Afghanistan, Pakistan, Somalia, Sudan), economic impact (Pakistan), and social and cultural acceptability (Ethiopia and Zanzibar). Both Somalia and Sudan used integrated service delivery as a criterion. In five countries, stakeholders were involved in the definition of decision criteria (Ethiopia, Pakistan, Somalia, Sudan and Zanzibar). In Pakistan, decision criteria were based on a policy document review, followed by survey among stakeholders and consultation in workshop (online supplemental box S2). Several countries reported on decision criteria in publicly available documents (online supplemental table S3).

## Step D: collect evidence on decision criteria for each service

Developing an EPHS should ideally be based on explicit criteria and the most updated local evidence available.^5 11^ As noted above, some of the most commonly used criteria included burden of disease, equity, FRP and CE. For illustrative purposes, online supplemental box S3 describes the use of local evidence in Afghanistan,^22^ and online supplemental box S4 describes the use of CE in the countries.

The governing body can organise a review of the quality of evidence by experts and/or stakeholders before it is used to prioritise services—our review shows that all countries have such a mechanism in place. Countries are generally advised to make public the evidence used in defining the EPHS.^11^ Most countries in our review shared the evidence either on a website, in a report or in a document sent to stakeholders (online supplemental table S4).

## Step E: prioritise services

In the appraisal step, the advisory committee interprets the results of the assessment in a broad perspective and then formulates recommendations for decision-makers. Governing bodies can best present evidence in a way that is easily accessible and understandable by the advisory committee.^10^ Subsequently, deliberation/discussion can be used as a way of interpreting this evidence and developing social and scientific judgements. The central challenge in these deliberations is to trade-off the different decision criteria.

A performance matrix can be a useful starting point—this simply presents the performance of a service against the decision criteria.^23^ There are different options for how advisory committees can interpret this matrix. First, they can undertake a qualitative approach, which simply involves deliberating on the performance matrix using explicitly defined criteria. Second, they can adopt a quantitative approach that is typically referred to as a multicriteria decision analysis using scoring and weighting techniques. However, in practice, this approach has important methodological challenges such as the neglect of the principle of opportunity costs.^23^ Third, they can use an approach with decision rules interpreting the performance matrix using a set of simple rules, for example, first ranking services on the basis of CE and then using deliberations to assess whether other criteria may affect the ranking. Irrespective of the approach, countries may always want to include a deliberative component in their appraisal process and to report on decisions, including argumentation, in a publicly available document.^23^ Our review showed that five countries (Afghanistan, Ethiopia, Pakistan, Somalia and Zanzibar) used a qualitative approach, and one country (Sudan) used a combined qualitative and quantitative approach (online supplemental table S5). All countries used deliberation in these approaches.

### Other aspects of prioritisation

Stakeholders involved in the prioritisation of services need to have the necessary capacity and be well trained for the task at hand.^24^ All focus countries in our review have involved a wide range of stakeholders in prioritising services. In addition, it is generally recommended that countries consider the available fiscal space in the prioritisation of services.^5^

While, for the sake of fairness, reimbursement decisions are ideally reached by consensus this is not always feasible because stakeholders may, for a variety of reasons, continue to disagree. The advisory committee can also reach a decision by majority voting where consensus is not otherwise achievable.^10^ In our analysis, all countries aimed to reach consensus, and in Pakistan, majority voting was used when consensus was not otherwise achieved.

In no country were committee meetings conducted in public. Only in Afghanistan the recordings/proceedings of the committee meetings were made available to the public. In all countries, the prioritisation process was described in publicly available documents.

## Step F: integrate implementation planning into EPHS revision

Countries can establish a plan that describes how services are implemented in terms of various health system aspects, such as copayments, delivery platform, health system barriers and required investments. They may want to make special efforts to secure an integrated service delivery, that is, to include foundational services in the package for undifferentiated conditions such as cough or fever, and to coordinate services across different levels of the health system to foster continuity of care. Such a plan can be developed in conjunction with stakeholders and described in a publicly available document. In our review, four countries (Ethiopia, Pakistan, Somalia and Zanzibar) established an implementation plan as an integral part of their EPHS revision (online supplemental table S6 and online supplemental box S5). In most countries, copayments, delivery platforms, health systems barriers and investments were taken into account, and in five countries, stakeholders were also involved. Five countries made the implementation plan publicly available.

## Step G: communication and appeal

Communication and appeal are important features that enhance the legitimacy of decision making by making the decision and underlying argumentation public. It is generally advised that countries should strive to ensure that EPHS coverage decisions are communicated to all relevant stakeholders, using a variety of channels.^11^ Our analysis showed that all countries had communication strategies in place to inform stakeholders.

‘Appeal’ refers to the need for a mechanism that gives stakeholders the possibility to apply for a revision of a decision, or by providing (new) arguments or evidence and receive a reasoned response.^14^ Countries can establish a protocol for appeal, including the requirements regarding provision of new evidence and clear revision rules. Our analysis shows that various countries had appeal mechanisms in place (online supplemental table S7).

## Institutionalisation

Countries had varying experiences regarding institutionalisation of their decision-making process (online supplemental table S8 and online supplemental box S6 for an example on Sudan). While most countries demonstrated a high political will for ongoing EPHS revision, only Ethiopia established this through regulation. Most countries designated a governing institute for EPHS revision. In addition, countries had recently revised their EPHS and most countries, therefore, had a good description of the decision-making process. This nevertheless needs to be endorsed as an established procedure in the health system and described in a formal document.

## Conclusion

In this paper, we have reviewed the experiences of six countries in terms of their decision-making processes for EPHS revision. Our analytical framework on the practical organisation distinguished several distinct steps and found that all countries appeared to have applied these. This confirms the relevance and validity of the framework and we advise countries embarking on a similar exercise to follow the same stepwise approach in shaping their decision-making process.

The steps, however, should not be considered as prescriptive or formulaic, and countries are encouraged to adapt the number, order and contents of steps to fit their own decision-making context. In our review, countries indeed differed in their implementation of various steps, for example, on the use of sub-committees to support the central advisory committee. Countries can learn from each other and select best practices accordingly.

Likewise, countries shared many characteristics on how they promoted the fairness of their decision-making process. For example, all countries organised some form of stakeholder involvement, although its practical implementation differed in terms of (A) number of stakeholders involved (Ethiopia involved no less than 80 stakeholders), (B) type of stakeholders involved (Zanzibar sets a nice example on patient and community involvement) and (C) mode of involvement (Pakistan allowed all stakeholders to fully participate in meetings, with voting power). Meaningful stakeholder involvement is key to fair decision-making processes, and we advise countries to prioritise this aspect when revising their EPHS development or revision process. In addition, all countries were transparent in terms of at least some of the steps of their decision-making process, for example, on the governance structure or on the decision criteria. We advise countries to be attentive to the need for transparency in all steps and describe these in publicly available documents. Where necessary, proactive efforts to inform stakeholders on the decision-making process may be required.

The review on institutionalisation shows that all six countries had a high degree of political will, an institution to pursue the work, the required capacity and an explicit prioritisation process. In addition, financial resources, either from domestic sources or development aid, were secured. However, in the most cases, the work was considered a project and not an ongoing activity embedded in the country’s health system. Countries are strongly advised to foster the institutionalisation of their EPHS development/revision process.^12^

All six countries were successful examples of EPHS development and revision. There have been other countries where, despite initial intentions, the process of defining or revising the EPHS has not yet started or, if it has begun, has not led to the final list of services as a package. Therefore, this paper only reviewed successful experiences and did not cover lessons learnt from possible failures.

## Supplementary material

10.1136/bmjgh-2022-010704 online supplemental file 1

10.1136/bmjgh-2022-010704 online supplemental file 2

10.1136/bmjgh-2022-010704 online supplemental file 3

10.1136/bmjgh-2022-010704 online supplemental file 4

10.1136/bmjgh-2022-010704 online supplemental file 5

10.1136/bmjgh-2022-010704 online supplemental file 6

10.1136/bmjgh-2022-010704 online supplemental table 1

10.1136/bmjgh-2022-010704 online supplemental table 2

10.1136/bmjgh-2022-010704 online supplemental table 3

10.1136/bmjgh-2022-010704 online supplemental table 4

10.1136/bmjgh-2022-010704 online supplemental table 5

10.1136/bmjgh-2022-010704 online supplemental table 6

10.1136/bmjgh-2022-010704 online supplemental table 7

10.1136/bmjgh-2022-010704 online supplemental table 8

## Data Availability

The data supporting the findings of this study are available upon request to the corresponding author.
