# Supplementary material for: Decision-making processes for essential packages of health services: experience from six countries
Source: BMJ Glob Health. 2023 Jan 19;8(Suppl 1):e010704. doi: 10.1136/bmjgh-2022-010704 (PMC9853142; doi:10.1136/bmjgh-2022-010704)
Supplement: online supplemental file 1 [file bmjgh-2022-010704supp001.pdf]

Supplementary Box S1

Box S1. Country information template

A. Install an advisory committee

1. Have advisory committees, program area advisory committees, and/or technical working groups been established? Describe membership in terms of number and affiliation.
2. How were members selected?
3. Do members need to sign a conflict-of-interest form before they are installed?
4. How were stakeholders involved? Describe their involvement in terms of communication/consultation / participation without voting / participation with voting, and how this was organised in practice.
5. Did committee members / stakeholders receive training on the EPHS process? Describe.
6. Is the membership and recruitment process described in a publicly available document?
7. Has a link been established with Ministry of Health and Ministry of Finance. Describe.

B. Map and select services for evaluation

1. How did the country select services for evaluation, i.e. for detailed assessment on the basis of evidence (e.g. compare existing package to DCP high priority package)? Describe.
2. How many services were evaluated? Describe if this was limited to certain DCP program areas and/or delivery platforms.
3. Were stakeholders involved in selecting services for evaluation? If so, describe which stakeholder groups and how.
4. Is the selection of services for evaluation described in a publicly available document?

C. Define decision criteria for prioritisation of services

1. How were decision criteria defined? Please describe if this is based on e.g. survey, or identification of social values through document analysis, or a combination.
2. Were stakeholders involved in the definition of decision criteria? If so, describe which stakeholder groups and how.
3. Which decision criteria were identified?
4. Were decision criteria operationalised in a way that allows evidence collection on these criteria?
5. Are defined decision criteria described in a publicly available document?

D. Collect evidence on decision criteria for each service

(Note that, following the scope of the paper, we will not make an inventory of sources and methods of data collection, except for cost-effectiveness)

1. How was evidence on cost-effectiveness collected? Describe e.g. review and contextualisation process.
2. Was the quality of evidence reviewed by experts and/or stakeholders? If so, describe which experts / stakeholder groups and how.
3. Is the collected evidence available in a public document?

E. Prioritise services

1. How was evidence presented to advisory committee / technical working groups (e.g. through color-coded evidence briefs and/or summary tables)?
2. Did committee members deliberate on the prioritization of services? If so, describe whether there was a structured process to interpret the evidence and trade-off criteria (e.g. weighing and scoring, or first ranking on cost-effectiveness and then modify ranking in deliberations).

3. How was reference made to the overall available fiscal space? (were costs of prioritised services added up to a total budget, and was an explicit budget constraint used?)
4. Were feasibility concerns included in the prioritisation of services?
5. Were stakeholders involved in the prioritization of services. If so, which stakeholder groups and how (communication/consultation / participation without voting / participation with voting).
6. What is the mandate of the committee – to develop recommendations or to take decisions? If it is to develop recommendations – to whom was it presented for endorsement?
7. How did the committee come to a decision/recommendation? (e.g. consensus or voting).
8. Are the committee meetings public, and/or are recordings / proceedings available to the public?
9. Is the prioritization process described in a publicly available document?

F. Integrate implementation planning into benefit package design

1. Was an implementation plan established in terms of e.g. co-payments (user fees), delivery platform, health system barriers and required health system investments? Describe which health systems aspects were takin into account.
2. Were stakeholders involved in the development of the implementation plan? If so, which stakeholder groups and how?
3. Is the implementation plan described in a publicly available document?

G. Communication and appeal

1. What mode of communication was used to inform stakeholders (including the broad public) on the outcomes of the EPHS decision-making, if any?
2. Are appeal options available for stakeholders wishing to revise decisions, and are these options pro-actively communicated to stakeholders?
