# Supplementary material for: Decision-making processes for essential packages of health services: experience from six countries
Source: BMJ Glob Health. 2023 Jan 19;8(Suppl 1):e010704. doi: 10.1136/bmjgh-2022-010704 (PMC9853142; doi:10.1136/bmjgh-2022-010704)
Supplement: online supplemental file 2 [file bmjgh-2022-010704supp002.pdf]

Supplementary Box S2

Box S2: In the spotlight: Defining decision criteria in Pakistan

The selection and definition of decision criteria involved several steps. Firstly, the project team, with representatives from the Ministry of National Health Services Regulations and Coordination (MNHSRC) and academic institutes, carried out a review of national health policy documents to identify relevant criteria. Second, the identified criteria were matched to the criteria proposed in the international literature, for which a recent published review was used. Third, the project team further specified the criteria and their definitions for feedback and approval by members of the technical working groups (TWG). This led to the preselection of eight criteria (effectiveness, health gain for money spent, avoidable burden of disease by the intervention, budget impact, feasibility, equity, financial risk protection, and social and economic impact).

Fourth, the MNHSRC conducted a Likert scale survey in which they asked members of the TWG to indicate the importance they attached to these criteria, whether they believed any criteria were missing, and to provide any additional comments or suggestions. In total 52 TWG members responded (response rate 52%). Based on the survey results, and feedback following the first appraisal workshop, several of the criteria were redefined (mainly phrased more in laymen's language). Especially the cost-effectiveness criterion proved difficult for participants to grasp and was rephrased as 'health gain for money spent'. No additional criteria were suggested. While effectiveness was one of the eight original criteria it was not used during the prioritization exercise as the services subjected to deliberation and prioritization were all considered effective, being a requirement for their inclusion in the DCP3 list of recommended interventions.
