# Supplementary material for: Decision-making processes for essential packages of health services: experience from six countries
Source: BMJ Glob Health. 2023 Jan 19;8(Suppl 1):e010704. doi: 10.1136/bmjgh-2022-010704 (PMC9853142; doi:10.1136/bmjgh-2022-010704)
Supplement: online supplemental file 3 [file bmjgh-2022-010704supp003.pdf]

Supplementary Box S3

Box S3: In the spotlight: Vernacular evidence in Afghanistan

The revision of the Afghanistan health priority package in 2018-2021 generated a particular mix of knowledge sharing and information use, creating a bank of evidence in a way that was unique to this particular development process. Contexts and processes really shape "vernacular evidence" that are produced in a specific place and at a specific time. Evidence is analysed, interpreted, discussed leading to debates producing new understandings and parameters to light. Keen and careful judgement was applied by experts, taking the micro and macro levels of the health system into account, guided by their experience and the dynamics within the group. In humanitarian response in particular, decision-makers will have to use their professional judgement "amidst the uncertainty of whether the existing research evidence can be applied to their unique setting" (Khalid et al. 2020). The IPEHS development is a good illustration of production of vernacular evidence, where the decision-making surrounding available evidence often came down to discussions and experience, rather than published material. At times vernacular evidence was a compromise in light of absence of specific evidence, and shaped by consensus building, shared ethics and morality. It is not less robust than other evidence, in fact, one could suggest that through its adaptations it is more explicit and tailored to the situation at hand. However, despite the "social" vetting of vernacular evidence, it is still at the mercy of authority, of those at the table with the most power.

Adapted from Lange I. et al., The development of Afghanistan's Integrated Package of Essential Health Services (IPEHS): Vernacular evidence, expertise and ethics in a priority setting process, Health Policy and Planning 2022

Reference: Khalid, A. F., Lavis, J. N., El-Jardali, F., & Vanstone, M. (2020). Supporting the use of research evidence in decision-making in crisis zones in low-and middle-income countries: a critical interpretive synthesis. Health research policy and systems, 18(1), 1-12
