# Supplementary material for: Decision-making processes for essential packages of health services: experience from six countries
Source: BMJ Glob Health. 2023 Jan 19;8(Suppl 1):e010704. doi: 10.1136/bmjgh-2022-010704 (PMC9853142; doi:10.1136/bmjgh-2022-010704)
Supplement: online supplemental file 4 [file bmjgh-2022-010704supp004.pdf]

Supplementary Box S4

Box S4: Using cost-effectiveness analyses for prioritisation and benefit package design

Cost-effectiveness analysis (CEA) is a popular criterion as it provides a useful way of maximising health gains within a budget constraint; however, generating local CEA data can be extremely resource-intensive, especially for LMICs (Glassman et al., 2016)

We reviewed the experiences of six LMICs in developing evidence for CEA using a short questionnaire, and found that given the lack of context-specific CEA studies, countries used one of three approaches to estimate CEA:(1) using existing tools and software to generate CEA estimates, (2) transferring existing evidence on CEA from similar health systems or global databases, and (3) using expert opinion to estimate value for money. For the first approach, whereby CEA estimates are generated using tools, some of the tools used predominantly in Zanzibar, Ethiopia and Somalia, includes the fair choices tool, the WHO-CHOICE tool, and the UHC Compendium respectively (Eregata et al., 2020, 2021). Other countries such as Pakistan and Afghanistan used the second approach, and transferred evidence on incremental cost-effectiveness ratios (ICERs) from global databases like DCP-3, or evidence from countries with the most similar health systems. Additionally, expert opinion and contextualization of published CEA evidence has been also applied in the Ethiopian case (Hailu et al., 2021) A third, slightly different approach was used by Sudan, where the team used expert opinion, as local and international experts provided a rating for each intervention based on its expected value for money.

Overall, it was clear that all countries considered CEA to be an important criterion, but none of the selected countries had an institutionalized process for generating local CEA data. It was interesting to note that countries used different approaches to incorporate CEA, given the lack of locally available evidence on this criterion. Each approach has its own strengths and limitations; for instance, using tools to generate local values for CEA helped provide local and updated estimates, but developing these estimates was still a resource-intensive process, made more challenging by the limited availability of primary data. The second approach to transfer evidence on CEA may be less time-consuming, but raises questions about the generalizability of the ICERs that were used. The third approach using expert opinion is likely the quickest, but has the obvious limitation of relying on the judgement of individuals. Given this variation of approaches, further work comparing these methodologies and tools to generate local evidence on CEA will be useful to provide guidance for LMICs looking to incorporate CEA in their prioritisation process.

References

- Eregata, G. T., Hailu, A., Stenberg, K., Johansson, K. A., Norheim, O. F., & Bertram, M. Y. (2021). Generalised cost-effectiveness analysis of 159 health interventions for the Ethiopian essential health service package. *Cost Effectiveness and Resource Allocation*, *19*(1), 2.
- Glassman, A., Giedion, U., Sakuma, Y., & Smith, P. C. (2016). Defining a Health Benefits Package: What Are the Necessary Processes? *Health Systems & Reform*, *2*(1), 39–50.
- Pitt, C., Vassall, A., Teerawattananon, Y., Griffiths, U. K., Guinness, L., Walker, D., Foster, N., & Hanson, K. (2016). Foreword: Health Economic Evaluations in Low- and Middle-income Countries: Methodological Issues and Challenges for Priority Setting. *Health Economics*, *25*, 1–5.
- World Health Organization. (2021). *Principles of health benefit packages*.
