# Supplementary material for: Decision-making processes for essential packages of health services: experience from six countries
Source: BMJ Glob Health. 2023 Jan 19;8(Suppl 1):e010704. doi: 10.1136/bmjgh-2022-010704 (PMC9853142; doi:10.1136/bmjgh-2022-010704)
Supplement: online supplemental file 5 [file bmjgh-2022-010704supp005.pdf]

Supplementary Box S5

Box S5: In the spotlight: Integrated service delivery approach in the revision of the Somalia's health service package

Frontline health workers deliver care across a range of conditions based on the demand of people. People seek health care for undifferentiated conditions, e.g., cough or fever, and not complaining about a specific disease such as pneumonia or tuberculosis. Therefore, listing interventions based on diseases (even if they have a high burden) might lead to a discrete package that will not be responsive to the demand of people. On the contrary, it should be clear what the health system does for each common demand, even if it is just a simple intervention, same as the provision of analgesics for pain relief due to particular cancer. The other issue is that services should be coordinated across different levels of the health care system and that the lower and higher-level services are aligned. For example, suppose simple lower respiratory infections are treated in the peripheral health centres, but if any complications appear, the case should refer to the hospital.

These two (i.e., the health system responds to the common demands of people and continuity of care across levels of service) are the main components of the integrated service delivery approach used in the revision of the health service package in Somalia. This approach provides a ground for the provision of people-centred services and has the following characteristics: addresses the way people present; includes all high-burden conditions; makes it easier for the user to understand what services are covered and where they are delivered; and ensures people move across the health system and that referral is coordinated with higher-level services.
