# Supplementary material for: Decision-making processes for essential packages of health services: experience from six countries
Source: BMJ Glob Health. 2023 Jan 19;8(Suppl 1):e010704. doi: 10.1136/bmjgh-2022-010704 (PMC9853142; doi:10.1136/bmjgh-2022-010704)
Supplement: online supplemental file 6 [file bmjgh-2022-010704supp006.pdf]

Supplementary Box S6

Box S6: In the spotlight: Arrangements for institutionalisation of service package in Sudan

In Sudan, a specific document was prepared for institutionalisation alongside the development of service package (Essential Health Benefits Package: EHBP). This document aimed to suggest a set of governance conventions, management actions, and resources needed to "institutionalise" the EHBP and related financial mechanisms from 2020 to 2025. The distinctive feature of this document is that the EHBP will ultimately be compatible with the broader governance of Sudan's health system.

As the method of developing this document, all essential functions and activities needed through the five-year period were identified. Second, the governance arrangements required for these functions and activities were mapped. Finally, advisory groups and technical panels were defined, as required.

The result was a board of national healthcare (chaired by the Federal Minister of Health (MoH), co-chaired by the Federal Minister of Labour and Social Development) for the governance and three subordinate boards for delivery, financing, and policy issues. In addition, EHBP activities will be coordinated by a dedicated EHBP programme team that gets inputs from experts' panels. The panels cover various EHBP development areas such as education and training or monitoring and evaluation.

The responsible bodies for implementation were also defined. The National Health Insurance Fund will hold and disburse "pooled healthcare funds". The Federal and States MoH will cover the sustainable delivery of the EHBP by government-owned health resources, and or in partnership with the private or third sector, and meeting standards and targets for efficacy, safety and values.
