# Supplementary material for: Decision-making processes for essential packages of health services: experience from six countries
Source: BMJ Glob Health. 2023 Jan 19;8(Suppl 1):e010704. doi: 10.1136/bmjgh-2022-010704 (PMC9853142; doi:10.1136/bmjgh-2022-010704)
Supplement: online supplemental table 1 [file bmjgh-2022-010704supp007.pdf]

Table S1: Summary of country experiences on installing an Advisory Committee (step A)

Indicator	Afghanistan	Ethiopia	Pakistan	Somalia	Sudan	Zanzibar (Tanzania)
What is of the composition of the Advisory Committee?	*National advisory group* with four members: Minister of Public Health, deputy Minister, Director of Information and Evaluation, Director of Health Financing and Economics with support from a group of int experts	National advisory group with 30 members, Regional advisory group with 36 members (3 from each region)	*National advisory committee (NAC)*[2] with 90 members: health professionals, development partners, provincial representation	Intergovernmental committee (7 members: Fed. Minister of Health, State Ministries of Health, advisors, academics, the private sector and international experts from international health partners	Supervisory committee (5 reps. from National Health Insurance Fund and Federal Ministry of Health	*National advisory team* with 15 members from Ministry of Health, Ministry of Finance, tertiary hospital representative, and Office of Chief Government Statistician
Was the advisory committee supported by sub-committees which developed preparatory recommendations on specific disease programs?	Yes. Nine working groups constituted in Afghanistan, including x representatives from MoPH, development partners and provincial directors	Yes. Nine working groups including 80 representatives from (subject matter experts from primary, secondary, and tertiary level, academia, MoH, regional health bureau, and development partners)	Yes. Four TWGs including a total of 183 members	No	Yes. One technical working group, consisting of 9 representatives from the National health Insurance Fund and Federal Ministry of Health and 2 representatives from WHO	Yes. 6 Technical working groups including health professionals (50), program managers (45), and representatives from civil society organisations including patient organizations (55), local government authorities (45), development partners (15), and MoH (15)
Was technical support provided?	By MoPH staff, international experts, WHO, international academic institutes, and international development partners	By international academic institutes WHO, Harvard School of Public Health, and Addis Ababa University	By project team (*UHC-BP secretariat)*, including staff from MoH and (inter)national academic institutes	By MoH technical working groups, and a task force including national and international development partners	By a project team comprising international experts in health economics and UHC development, and 13 clinical expert teams comprising Sudan health professionals	By international academic institutes and project team (*core team*) of 12 members from MoH, MoF and Office of Chief Government Statistician
Did the (sub)committee involve patients or patient representativeness?	No	No[1]	No	No	No	Yes
Did the (sub)committee involve public representatives?	No	No	No	No	No	Yes
How were stakeholders involved in the (sub)committee?	Consultation and participation in deliberations but without voting rights	Participation in deliberations	Participation in deliberations, with voting power	Stakeholders (representatives from service providers, policy makers, purchasers, financiers, academia and private sector) through a	Consultation and participation in deliberations but without voting rights.	Consultation and participation in deliberations

[1] Patient representatives (for cancer, chronic kidney disease, diabetes mellites) were involved in step C (Map and select services for evaluation) and D (Define decision criteria for prioritization of services).

[2] In addition, a Steering Committee (SC) was established involving stakeholders who reported to the Minister of Health. The National Advisory Committee reported to the SC.

				steering committee were involved in the service package development. Voting mechanism was not part of the process		
Did members declare conflict-of-interest?	No	No	Yes	No	No	No
Is the membership and recruitment process described in a publicly available document? If yes, how (report, website)?	Yes	Yes, Direct email communication and an official letter from the Office of the Minister sent to stakeholders	Yes, in a report	Yes, through a letter sent to all stakeholders	Yes, in a report. Only in summary and not including the recruitment process	Yes, in a report
Were stakeholders involved in still other ways, i.e. outside the mentioned sub-committees?	Yes, they were consulted to review the final version of the IPEHS	Yes	No	Yes, stakeholders have reviewed the service package before it was endorsed by the Ministry of Health	No	Yes

Abbreviations: IPEHS=Integrated Package of Essential Health Services; MoF=Ministry of Finance; MoH = Ministry of Health; MoPH = Ministry of Public Health; NAC=National Advisory Committee; TWGs=Technical Working Groups; UHC-BP=Universal Health Coverage-Benefit Package; WHO=World Health Organization
