# Supplementary material for: Decision-making processes for essential packages of health services: experience from six countries
Source: BMJ Glob Health. 2023 Jan 19;8(Suppl 1):e010704. doi: 10.1136/bmjgh-2022-010704 (PMC9853142; doi:10.1136/bmjgh-2022-010704)
Supplement: online supplemental table 2 [file bmjgh-2022-010704supp008.pdf]

Table S2: Summary of country experiences on mapping and selecting services for evaluation (Step B)

Indicator	Afghanistan	Ethiopia	Pakistan	Somalia	Sudan	Zanzibar (Tanzania)
Which model package was used as starting point?	DCP3 HPP	DCP3 (EUHC or HPP?), WHO-CHOICE	DCP3 EUHC	DCP3 EUHC expanded with service listing in UHC Compendium	DCP3 EUHC expanded with WHO-EMRO UHC-Priority Benefit Package	DCP3 EUHC
Was this model package compared to the existing package?	?	Yes, to the 2005 Essential Health Service Package (ESPH)	Yes it was compared to the existing packages	Yes	Yes	Essential Health Care Package (EHCP)
Were all services evaluated or only a selection?	All services	All services	All services	All services	All services	All services
Were services assessed on their relevance?	Yes. Based on BoD	Yes. Based on BoD	Yes	Yes. Based on the common undifferentiated problems and BoD	Yes	Yes
Were stakeholders involved in the selection of services?	National advisory group and Expert Committee members were involved	Yes, committees	Four technical working group were involved	Yes, experts, donors and providers from public and the private sector	The 13 expert clinical committees	The six technical working groups were involved
Is information on selection of services publicly available? If yes, how (report, website)?	Yes	Yes	Yes, publicly available on website	Not publicly accessible	Yes, available at https://sudan-ehbp.com/essential-health-benefits-package	Report

Abbreviations: BoD = Burden of Disease; DCP3=Disease Control Priorities 3; HPP=highest priority package; EHCP=Essential Health Care Package; EMRO= Eastern Mediterranean Regional Office; ESPH=Essential Health Service Package; UHC=Essential UHC package; WHO=World Health Organization
