# Supplementary material for: Decision-making processes for essential packages of health services: experience from six countries
Source: BMJ Glob Health. 2023 Jan 19;8(Suppl 1):e010704. doi: 10.1136/bmjgh-2022-010704 (PMC9853142; doi:10.1136/bmjgh-2022-010704)
Supplement: online supplemental table 3 [file bmjgh-2022-010704supp009.pdf]

Table S3: Summary of country experiences on defining decision criteria for prioritisation of services (Step C)

Indicator		Afghanistan	Ethiopia	Pakistan	Somalia	Sudan	Zanzibar (Tanzania)
Selected criterion*	Burden of disease		√		√	√ (Meets health need)	√
	Effectiveness	√		√ (Avoidable burden)		√	
	Quality of evidence			√		√	
	Financial Risk Protection	√ (Affordability)	√	√			√
	Equity	√	√	√			√
	Cost-effectiveness		√	√ (Health gain for money spent)	√ (Likely value for money)	√ (Likely value for money)	√
	Budget impact		√	√	√ (Affordability)	√	√
	Integrated service delivery				√	√	
	Feasibility	√		√	√	√ (to inform potential timing)	
	Socio-economic impact			√			
	Public and political acceptability		√		√ (Political acceptability)		√
How were decision criteria defined and by whom?		National advisory group and International expert group	Literature review, followed by deliberation among MoH leadership and all stakeholders. Final list of criteria was based on decision by MoH.	Policy document review, followed by survey among stakeholders and consultation in workshop	Proposed by expert group followed by stakeholder consultation (MoH staff, program managers and service providers, international partners)	Decision process, criteria and weighting selected in a workshop with the Technical Group and other ministry stakeholders.	Proposal from deliberative meetings were discussed and final list decided by the executive committee of the MOH
Were stakeholders involved in this step?		No	Yes	Yes	Yes	Yes	Yes
Is information on process and criteria definitions publicly available? If yes, how (report, website)?		Yes (how?)	Yes, in report	Yes, in report	The process of work published and endorsed by the MoH	Yes available at https://sudan-ehbp.com/essential-health-benefits-package	..

* The names of the criteria used by the countries were interpreted in terms of common criteria definitions. The original naming of the criterion is provided in between brackets.
