# Supplementary material for: Decision-making processes for essential packages of health services: experience from six countries
Source: BMJ Glob Health. 2023 Jan 19;8(Suppl 1):e010704. doi: 10.1136/bmjgh-2022-010704 (PMC9853142; doi:10.1136/bmjgh-2022-010704)
Supplement: online supplemental table 4 [file bmjgh-2022-010704supp0010.pdf]

Table S4: Summary of country experiences on evidence collection (Step D)*

Indicator	Afghanistan	Ethiopia	Pakistan	Somalia	Sudan	Zanzibar (Tanzania)
Was evidence reviewed before it was used for decision-making?	Yes	Yes	Yes	The evidence was collected from trusted global sources and so considered as reliable.	Yes	Yes
Were stakeholders involved in the collection and review of evidence?	The international expert group and national working groups were involved	Committees and TWGs established at different levels were involved	TWG members were engaged in review of service descriptions and collected evidence	All relevant stakeholders including MOH and partners/donors were involved	Yes, 13 expert groups were engaged to review all services	Yes, all TWGs were engaged in the review of the evidence
Is information on this step publicly available? If yes, how (report, website)?	Yes, on MoPH website	Yes, report	No	The data sources and criteria used are elaborated in a document shared with stakeholders	Not yet but it will be published once the final selection is complete	Yes, report

Abbreviations: MOH=Ministry of Health; MoPH = Ministry of Public Health; TWGs=Technical Working Groups
