# Supplementary material for: Decision-making processes for essential packages of health services: experience from six countries
Source: BMJ Glob Health. 2023 Jan 19;8(Suppl 1):e010704. doi: 10.1136/bmjgh-2022-010704 (PMC9853142; doi:10.1136/bmjgh-2022-010704)
Supplement: online supplemental table 5 [file bmjgh-2022-010704supp0011.pdf]

Table S5: Summary of country experiences on prioritisation of services (Step E)

Indicator	Afghanistan	Ethiopia	Pakistan	Somalia	Sudan	Zanzibar (Tanzania)
Did the committee use a structured approach to prioritise services?	Qualitative approach – deliberations on the basis of explicit criteria.	Qualitative approach – deliberations on the basis of explicit scored criteria	Qualitative approach – deliberations on the basis of explicit scored criteria	Qualitative approach – deliberations on the basis of explicit criteria	Combined qualitative and quantitative approach, including scoring and weighing as starting point for deliberation	Qualitative approach – deliberations on the basis of explicit criteria
Did the committee deliberate to prioritise services?	Yes	Yes	Yes	Yes	Yes	Yes
Did the committee take into account the required budget and available budget (fiscal space) when prioritising services?	The required budget was estimated but not the fiscal space	Yes, in fiscal space	Yes, both required budget and available fiscal space	The required budget was estimated but not the fiscal space	The required budget was estimated but fiscal space was not	Projection for the coming 10 years was made and fiscal space will be done using the FairChoice tool
Did the committee take into account feasibility concerns when prioritising services?	Yes	Yes	Yes	Yes	Yes – in relation to timing	Yes
Were stakeholders involved in prioritising services?	Yes, through all committees as mentioned in Appendix Table 1	Yes	Yes, through all committees as mentioned in Table 1	Yes, through all committees as mentioned in Table 1	Yes, through all committees as mentioned in Table 1	Yes, through all committees as mentioned in Table 1
How did the committee come to a decision?	Consensus	Consensus	TWG members voted on classification of services as low, medium or high priority. If consensus was not achieved, majority vote was used. In NAC and SC consensus was used	Consensus	Consensus	Consensus
How was evidence presented to the committee?	Analysis reports with summaries and excel sheet	Draft report and interactive excel sheet	Evidence sheets which colour coded evidence on the criteria burden of disease, budget impact and cost-effectiveness	Results from the Somalia Health & Demographic Survey 2020, colour coded matrix of the burden of disease analysis and resources mapping and expenditure tracking analysis	Excel sheet	The FairChoices model was used to present evidence on cost-effectiveness, budget impact and health benefit gains in low, moderate and high performance for each delivery platform
Are the committee meetings public?	No	No	No	No	No	No
Are recordings and/or proceedings of the committee meetings available to the public?	Yes	No	No	No	No	No
Is the prioritisation process described in a publicly available document? If yes, how (report, website)?	Yes (how?)	Yes, on a website	Yes, in a report	Yes, in a report available online. The prioritisation has not been finalised yet	Yes, available at https://sudan-ehbp.com/essential-health-benefits-package	Yes, it was well described in the report

Abbreviations: NAC=National Advisory Committee; SC=Steering Committee; TWG=Technical Working Group
