# Supplementary material for: Decision-making processes for essential packages of health services: experience from six countries
Source: BMJ Glob Health. 2023 Jan 19;8(Suppl 1):e010704. doi: 10.1136/bmjgh-2022-010704 (PMC9853142; doi:10.1136/bmjgh-2022-010704)
Supplement: online supplemental table 6 [file bmjgh-2022-010704supp0012.pdf]

Table S6: Summary of country experiences on development of implementation planning (Step F)

Indicator	Afghanistan	Ethiopia	Pakistan	Somalia	Sudan	Zanzibar (Tanzania)
Was an implementation plan developed?	No, due to the arrival of Taliban	Yes	Yes	Yes, the implementation plan was developed as part of the EPHS	No clear implementation plan, though the recommendations are in place	Yes
Were levels of co-payment taken into account?	Yes	Yes	No	The rollout of the package will be done incrementally depending on the available resources and capacity.	Not yet	Currently Zanzibar offers health care services free of charge
Were delivery platform taken into account?	Yes	Yes	Yes	Yes, the package targeted to offers services at all levels	Yes the package explicitly includes delivery platform	Yes
Were health system barriers taken into account?	Yes	Yes	Yes	Yes, this includes consideration of nomadic populations, IDPs, and insecure areas of the country	Yes, in terms of current coverage.	Yes
Were health system investments taken into account?	Were supposed to be calculated (but?)	Yes	Yes, as a percentage of costs of the package	Yes, and the investment case for the health sector was developed with main focus on service delivery and prioritised health system strengthening provisions.	Not yet	Yes
Were stakeholders involved in developing the implementation plan?	Yes	Yes	Yes, through their membership of the Technical Working Groups	Yes, through their membership of the the task force	Not yet	Yes, the plan was developed by core team and shared with experts and program managers for their input
Is the implementation plan (becoming) available in a public document? If yes, how (report, website)?	No	Yes, in report	Yes, on website	Yes, in report	Yes, available at https://sudan-ehbp.com/essential-health-benefits-package	Yes, in report

Abbreviations: EPHS=Essential Package of Health Services; IDPs=internally displaced persons
