# Supplementary material for: Decision-making processes for essential packages of health services: experience from six countries
Source: BMJ Glob Health. 2023 Jan 19;8(Suppl 1):e010704. doi: 10.1136/bmjgh-2022-010704 (PMC9853142; doi:10.1136/bmjgh-2022-010704)
Supplement: online supplemental table 7 [file bmjgh-2022-010704supp0013.pdf]

Table S7: Summary of country experiences on communication and appeal (Step G)

Indicator	Afghanistan	Ethiopia	Pakistan	Somalia	Sudan	Zanzibar (Tanzania)
What mode of communication was used to inform stakeholders on the outcomes of the EPHS decision-making?	Consultation	Official letter, email, public launching, media release, and press release	Official letter, email, Steering Committee meeting/ Inter-ministerial forum, public report, press release	Through health sector coordination meetings, formal launching with stakeholders and media participation, emails and reports shared directly and through MoH website	The EPHS is not yet finalised / approved	Through consultative meetings
Were appeal options available for stakeholders wishing to revise decisions	Through two consultation rounds in 2021	Yes, appeal can be made to the Executive Committee of the Ministry of Health	Yes. Provinces were given opportunity to revise the decisions at national level	Was not part of the process	Not yet.	No
Were appeal options pro-actively communicated to stakeholders?	Yes	Yes	Yes	No	Not yet.	No

Abbreviations: MoH=Ministry of Health
