# Supplementary material for: Decision-making processes for essential packages of health services: experience from six countries
Source: BMJ Glob Health. 2023 Jan 19;8(Suppl 1):e010704. doi: 10.1136/bmjgh-2022-010704 (PMC9853142; doi:10.1136/bmjgh-2022-010704)
Supplement: online supplemental table 8 [file bmjgh-2022-010704supp0014.pdf]

Table S8: Summary of country experiences on institutionalisation

Indicator	Afghanistan	Ethiopia	Pakistan	Somalia	Sudan	Zanzibar (Tanzania)
Is there an explicit requirement (e.g., legal framework) in place that ensures the use of ongoing EPHS revision in the country?	The political will existed, evident from a president's letter but not enforced as an explicit obligation	Yes. There is a resolution and legal framework in place	The concept is elaborated in upstream documents as policy but not as an explicit requirement	Yes, while it is a decision by the Minister of Health, it is still not a written mandatory requirement	Stated by directorates of the Minister of Health, but not yet translated in a formal requirement	There is no specific "explicit requirement" for the package
Is an institution designated for governing ongoing EPHS revision in the country?	Yes	Yes. The Federal Ministry of Health is solely designated	Responsibilities were clear at federal level. At the provincial level, local institutes will be designed.	The Ministry of Health is responsible for guiding and overseeing the revision through a participatory process	A governing structure has been proposed but not established yet	The is led by the Ministry of Health itself with technical supports. Strengthening of existing bodies has already started such as establishing a financing unit in the MOH
Is the ongoing EPHS revision process described in a formal document?	No	Yes. The process is described in a national document	The process is well defined but not described in a formal document	The Ministry of Health developed a concept note describing the revision process	The process is well defined but not described in a formal document	Yes, described in a report
Does this institute have sufficient funds for ongoing EPHS revision activities?	No	The MoH allocates some funding but this is not sufficient.	No	No	It is stated as an activity in the upcoming plan that can guarantee sufficient resources	It relies on development partners
Are there plans to build the required technical capacity?	No	Yes. Training on Health Economics at the Master and PhD level is ongoing	The Federal Ministry envisages the provinces. However, further technical capacity is needed at the federal level	There is no plan yet	It is proposed in the plan	Yes, a team of 12 members established and trained and PhD opportunities obtained
